# cPLA_2_α Enzyme Inhibition Attenuates Inflammation and Keratinocyte Proliferation

**DOI:** 10.3390/biom10101402

**Published:** 2020-10-02

**Authors:** Felicity J. Ashcroft, Nur Mahammad, Helene Midtun Flatekvål, Astrid J. Feuerherm, Berit Johansen

**Affiliations:** 1 Department of Biology, Norwegian University of Science and Technology, Realfagbygget, 7491 Trondheim, Norway; felicity.ashcroft@ntnu.no (F.J.A.); nurm@ntnu.no (N.M.); helene.flatekval@lyse.net (H.M.F.); astfe@tkmidt.no (A.J.F.); 2 Center for Oral Health Services and Research (TkMidt), 7030 Trondheim, Norway

**Keywords:** cPLA_2_α, psoriasis, proliferation, anti-inflammatory

## Abstract

As a regulator of cellular inflammation and proliferation, cytosolic phospholipase A_2_ α (cPLA_2_α) is a promising therapeutic target for psoriasis; indeed, the cPLA_2_α inhibitor AVX001 has shown efficacy against plaque psoriasis in a phase I/IIa clinical trial. To improve our understanding of the anti-psoriatic properties of AVX001, we sought to determine how the compound modulates inflammation and keratinocyte hyperproliferation, key characteristics of the psoriatic epidermis. We measured eicosanoid release from human peripheral blood mononuclear cells (PBMC) and immortalized keratinocytes (HaCaT) and studied proliferation in HaCaT grown as monolayers and stratified cultures. We demonstrated that inhibition of cPLA_2_α using AVX001 produced a balanced reduction of prostaglandins and leukotrienes; significantly limited prostaglandin E_2_ (PGE_2_) release from both PBMC and HaCaT in response to pro-inflammatory stimuli; attenuated growth factor-induced arachidonic acid and PGE_2_ release from HaCaT; and inhibited keratinocyte proliferation in the absence and presence of exogenous growth factors, as well as in stratified cultures. These data suggest that the anti-psoriatic properties of AVX001 could result from a combination of anti-inflammatory and anti-proliferative effects, probably due to reduced local eicosanoid availability.

## 1. Introduction

The phospholipase A_2_ (PLA_2_) superfamily of enzymes cleave phospholipids at the sn-2 position to release free fatty acids and lysophospholipids. These are the precursors to a multitude of lipid signaling molecules including the eicosanoids, which are metabolites of arachidonic acid (AA) and have important roles in inflammation and inflammatory diseases. Cytosolic phospholipase A_2_ α (cPLA_2_α) is the only PLA_2_ enzyme with high specificity for phospholipids carrying AA at the sn-2 position, placing it as an important upstream regulator of eicosanoid production [1]. When activated by extracellular stimuli, cPLA_2_α undergoes Ca^++^-dependent translocation from the cytoplasm to intracellular membranes and becomes predominantly localized to the peri-nuclear region of the cell [2,3,4]. This is where metabolism of AA by the cyclo-oxygenase (COX) and lipo-oxygenase (LOX) pathways typically occurs, producing prostaglandins and thromboxane A_2_ (TxA_2_), or leukotrienes, hydroxyeicosatetraenoic acids (HETEs), and hydroperoxyeicosatetraenoic acids (HPETEs), respectively. The importance of cPLA2α for stimulus-induced eicosanoid production and the pathogenesis of inflammation has been demonstrated by gene silencing both in vitro [5,6] and in animal models [7,8,9,10,11], and from the use of specific inhibitors of cPLA_2_α in preclinical models of inflammatory diseases, as was recently reviewed by Nikolaou et al. [12]. Examples include the use of the indole-derivative ZPL-5212372 in asthma and atopic dermatitis [13], the pyrrolidine-based compound RSC-3388 in a *Streptococcus pneumonia* infection model [14], and the ω-3 polyunsaturated fatty acid (PUFA) derivatives AVX001 and AVX002 in collagen-induced arthritis [15].

Plaque psoriasis (psoriasis vulgaris) is a disease with a chronic inflammatory phenotype that drives the hyperproliferation and aberrant differentiation of the epidermis [16]. Chronic inflammation in psoriasis is associated with higher expression of PLA_2_ enzymes [17,18,19] and increased levels of eicosanoids [20,21,22,23]. Evidence for the involvement of eicosanoids in psoriasis is supported by mouse models of the disease—the leukotriene B_4_ (LTB_4_) receptor 1 and TxA_2_ receptor have critical roles in imiquimod-induced skin inflammation [24,25,26], and prostaglandin E_2_ (PGE_2_) acting at prostaglandin receptors EP2 and EP4 is important for Th17-dependent inflammation in interleukin 23 (IL-23)-induced psoriasis [27]. Suppression of eicosanoid production is therefore an interesting prospect for treating psoriasis.

Non-steroidal anti-inflammatory drugs (NSAIDs) that inhibit AA metabolism via the COX pathway are commonly used for their analgesic, anti-inflammatory, and antithrombotic actions; however, their use is associated with many adverse gastrointestinal and cardiovascular effects (as reviewed in [28]) and they can induce or exacerbate psoriasis. The latter effect at least is postulated to be attributable to a skewed eicosanoid profile and the accumulation of leukotrienes (reviewed in [29,30]). Thus, it has been hypothesized that creating a more balanced suppression of eicosanoids using either dual COX-LOX inhibitors or by suppression of AA production using PLA_2_ inhibitors (reviewed in [12,31]) would provide a better and safer therapeutic option.

The cPLA_2_α inhibitor AVX001 is a ω-3 PUFA-derivative developed by Avexxin (now Coegin Pharma) that was demonstrated to be highly selective and to inhibit the in vitro activity of cPLA_2_α with an IC_50_ of 120nM, being more potent than either docosahexaenoic acid (DHA) or the ω-6 PUFA derivative arachidonyl trifluoromethyl ketone (AACOCF_3_, ATK) [15,32]. A topical application of AVX001 was trialed in a randomized, double-blind, placebo-controlled, dose-escalation first-in-man study to assess its safety and efficacy in patients with mild to moderate plaque psoriasis [33]. AVX001 showed significant efficacy and was well tolerated up to the maximum dose tested of 5%, supporting the targeting of cPLA_2_α as a safe therapeutic strategy. The specificity and potency of cPLA_2_α inhibition by AVX001 has been demonstrated [15,32], however, its mode of action in psoriasis remains to be determined. Given the documented role of cPLA_2_α in mediating inflammatory signals in monocytes and keratinocytes [34,35,36,37] and the more recent interest in cPLA_2_α as a driver of cellular proliferation [38], we sought to study potential modes of action of AVX001 in psoriatic skin by investigating its effects on inflammation and proliferation using human peripheral blood mononuclear cells (PBMC) and keratinocytes.

## 2. Materials and Methods

### 2.1. Materials

Cell culture media and chemicals were purchased from Sigma-Aldrich (St. Louis, MO, US) unless stated otherwise. A23178, naproxen, celecoxib calcipotriol hydrate, and lipopolysaccharide (LPS) were purchased from Sigma-Aldrich. Nordihydroguaiaretic acid (NDGA) was from Cayman chemicals (Ann Arbor, MI, USA) Recombinant human epidermal growth factor (EGF) and tumour necrosis factor (TNF)-α were from R&D systems (Abdingdon, UK). The fluoroketone AVX001 was synthesized and characterized according to Holmeide and Skattebol [39], and provided by Dr. Inger Reidun Aukrust and Dr. Marcel Sandberg (Synthetica AS, Oslo, Norway). AVX001 was stored at −80 °C as a 20 mM stock solution in dimethyl sulphoxide (DMSO) under argon gas to minimize oxidation.

### 2.2. PBMC Isolation and Treatment

Blood was recruited from healthy donors at St. Olavs Hospital HF, the Bloodbank (project approved by Regional Ethical Committee of Mid-Norway; #2016/553). Peripheral blood mononuclear cells (PBMC) were isolated using SepMate separation tubes with LymphoPrep density gradient medium from STEMCELL Technologies (Cambridge, UK), according to the manufacturer’s recommendations. For experiments, 1 x 10^6^ cells per well were plated in 1 mL Roswell Park Memorial Insitute (RPMI) medium supplemented with 5% fetal bovine serum (FBS), 0.3 mg/mL glutamine, and 0.1 mg/mL gentamicin. Inhibitors were added 2 h prior to the addition of the Ca^++^ ionophore A23178 (30 µM, 15 min) to activate cPLA_2_α or lipopolysaccharide (LPS) (10 ng/mL, 72 h) as a potent inducer of inflammation. Following treatment, the cell suspensions were centrifuged to isolate the supernatant from the cell fraction. Samples were stored at −80 °C until analysis.

### 2.3. Enzyme-Linked Immunoassay Detection of Eicosanoids

Cell supernatant samples were analyzed by enzyme-linked immunosorbent assay (ELISA) for PGE_2_ (Cayman #514435), LTB_4_ (Cayman #10009292), TxB_2_ (Cayman #501020), or 12S-HETE (Enzo Lifesciences #ADI-900-050) according to the manufacturers’ protocols. Cell supernatants were assayed at dilutions of 1:100 for PGE_2_, except supernatants from non-LPS-treated PBMC that were assayed undiluted in all assays. Supernatants were hybridized overnight, and the enzymatic conversion of the substrate was read at OD420 nm. Data were processed using a 4-parameter logistic fit model.

### 2.4. Culture of HaCaT Keratinocytes

#### 2.4.1. Maintenance

The spontaneously immortalized skin keratinocyte cell line HaCaT [40] was kindly provided by Prof. N. Fusenig (Heidelberg, Deutsches Krebsforschungszentrum, Germany). These cells are commonly used to study proliferative and inflammatory responses in psoriasis research [41,42,43,44,45,46], as they express epidermal growth factor receptor (EGFR) and can proliferate both independently of, as well as in response to, stimulation with growth factors [47]. HaCaT were maintained in Dulbecco’s modified Eagle Medium (DMEM) supplemented with 5% (*v*/*v*) FBS, 0.3 mg/mL glutamine, and 0.1 mg/mL gentamicin (DMEM-5) at 37 °C with 5% CO_2_ in a humidified atmosphere at sub-confluency to prevent differentiation. Treatments were carried out in DMEM supplemented with 0.5% (*v*/*v*) FBS and 0.3 mg/mL glutamine (DMEM-0.5)

#### 2.4.2. Eicosanoid Release

For analysis of eicosanoid release, we plated HaCaT in 12-well plates at 5 × 10^4^ cells per well in DMEM-5 and cultured them for 3 days until reaching approximately 50% confluency, when the media was replaced with DMEM-0.5. The following day, the cells were stimulated with tumour necrosis factor (TNF)-α (30 ng/mL, 72 h), EGF (30 ng/mL, 24 h), or calcipotriol (10 nM, 72 h).

### 2.5. [3H]-Arachidonic Acid Release Assay

At 2 days post-confluency, we labelled HaCaT for 18 h with ^3^H-AA (0.4 μCi/mL) in DMEM/0.5% FBS. After labelling, the cells were washed twice with phosphate-buffered saline (PBS) containing fatty acid-free bovine serum albumin (BSA) (2 mg/mL) in order to remove unincorporated radioactivity. After stimulation (EGF 100 ng/mL, 60 min), the supernatants were cleared of detached cells by centrifugation (13,000 rpm, 10 min). The release of ^3^H-AA from the cells was assessed by liquid scintillation counting in a LS 6500 Multi-Purpose Scintillation Counter (Beckman Coulter, Brea, CA, USA). Adherent cells were dissolved in 1M NaOH in order to determine incorporated ^3^H-AA in the cells by liquid scintillation counting. The results are given as released ^3^H-AA in the supernatants relative to total ^3^H-AA incorporated into the cells.

### 2.6. Resazurin Assay

HaCaT were seeded in 96-well plates in DMEM-5 at a density of 3000 cells per well. Following 72 h of cultivation, when cells reached a density of approximately 50%, we replaced the medium with DMEM-0.5. The following day, the cells were treated with AVX001, in a series of eight wells per treatment, for 24h. Resazurin (RnD systems, Abingdon, United Kingdom) was added according to the manufacturer’s instructions and left to incubate for 2h at 37 °C with 5% CO_2_ in a humidified atmosphere. Fluorescence was read at 544 nm excitation and 590 nm emission wavelengths using the Cytation 5 cell imaging multimode reader (Biotek Instruments, Winooski, VT, USA).

### 2.7. High Throughput Microscopy Assay for Population Analysis of Cell Cycle and Apoptosis

Cells were seeded in Greiner Bio-one CELLSTAR 96-well flat clear flat-bottomed plates (BioNordika, Oslo, Norway) in DMEM-5 at a density of 3000 cells per well. After 72 h, when cells reached a density of approximately 50%, we replaced the medium with DMEM-0.5. The following day, cells were treated with vehicle, AVX001, or etoposide (10 µM) in DMEM-0.5 for 24 h. We then followed the manufacturer’s guidelines for the Click-iT 5-ethyl-2′-deoxyuridine (EdU) Alexa Fluor 594 imaging kit (ThermoFisher Scientific, Waltham, MA, USA) using a final concentration of 10 µM EdU per well incubated for a further 2 h at 37 °C, 5% CO_2_. Following incubation with EdU, we removed the media and replaced it with the CellEvent Caspase 3/7 Green detection reagent (ThermoFisher Scientific) prepared at 2 µM in Dulbecco’s (D)-PBS +5% FBS. The cells were incubated for a further 45 min, then the reagent was removed, and the cells were immediately fixed using 4% formaldehyde in D-PBS for 20 min on ice. Permeabilization was carried out using 0.1% Triton-X 100 in D-PBS and the Click-iT reaction was performed according to the manufacturer’s guidelines using the Alexa-594 picoyl azide to label incorporated EdU. Finally, the cells were counterstained by incubation with 1 µg/mL 4′,6-diamidino-2-phenylindole (DAPI) (ThermoFisher Scientific) in D-PBS for 5 min. DAPI solution was removed and replaced with D-PBS for imaging. Plates were stored in the dark at 4 °C. All steps were performed at room temperature unless otherwise stated. Automated imaging was carried out on the Cytation5 cell imaging multimode reader (Biotek Instruments) at 4× magnification using DAPI, TexasRed, and GFP filter sets to image the DAPI, Alexa-594, and CellEvent Green signals, respectively. Four images were taken per well and 3 wells per treatment were used for the analysis.

Image analysis was performed in the freeware CellProfiler version 3.1.9 [48]. Firstly, nuclei were segmented from DAPI images using an Ostu 2-class thresholding approach, and were then counted. In further analyses, filters were employed to remove images with fewer than 50 cells. For cell cycle analysis, the total DAPI intensity and total EdU staining were then measured per nuclei from 12 images per treatment group and the freeware Flowing version 2.5.1 (Perttu Terho, Turku Centre for Biotechnology) was used to identify cells in G1, G2, and S-phases of the cell cycle, with gating based on log_10_ total EdU intensity vs. total DNA intensity. Apoptotic cells were identified on the basis of robust-background thresholding of the CellEvent Green signal and reported as a percentage of the total number of cells per image. Four images were taken per well and data were based on 3 wells per treatment group. To calculate the proliferative index, we segmented EdU-positive cells using an Ostu 2-class thresholding approach and reported them as a proportion of the total number of cells per image. Four images were taken per well and data were based on 3 wells per treatment group.

### 2.8. RNA Extraction and Real-Time Quantitative PCR

Cells were seeded in 6-well plates in DMEM-5. Following 72 h of cultivation, when cells reached a density of approximately 50%, we replaced the medium with DMEM-0.5. The following day, the cells were preincubated with AVX001 for 2 h prior to stimulation with EGF (30 ng/mL, 4 h). Total RNA was extracted with Total RNA kit I from Omega BIO-TEK (Norcross, GA, USA) according to the manufacturer′s protocol. The amount and purity of the RNA samples were quantified using a Nanodrop One/One^C^ Microvolume UV–VIS Spectrophotometer (ND-ONE-W) from ThermoFisher Scientific. RNA samples with absorbance (A) A260/A230 between 1.8 and 2.1 and A260/280 between 2.0 and 2.2 were accepted. Reverse transcription was carried out using the QuantiTect Reverse Transcription Kit (Qiagen, Hilden, Germany) with 1 µg of RNA per sample, according to the manufacturer’s protocol. Real-time PCR analysis was performed using the LightCycler 480 SYBR Green I Master MIX and LightCycler 96 instrument from Roche (Basel, Switzerland), according to the manufacturer’s protocol.

### 2.9. 3D Culture of HaCaT Keratinocytes

3D stratified HaCaT cultures were grown in Nunc cell culture inserts (0.4 µm pore size) using the 24-well carrier plates system (Thermo Fisher Scientific #141002) The culture inserts were coated using the Coating Matrix kit (Thermofisher Scientific #R-011-K) according to the manufacturer’s protocol. HaCaT were plated at a density of 0.3 × 10^5^ cells per insert in 0.5 mL DMEM-5 and incubated for 24 h before being lifted to the air–liquid interface. The media in the lower chamber was replaced with DMEM-5 (without antibiotics) + 1 ng/mL EGF, and 5 µg/mL L-ascorbic acid in the absence or presence of AVX001 (5 µM). The media in the lower chambers and treatments were changed every 3rd day for 12 days.

The cultures were fixed in 4% paraformaldehyde (PFA) overnight, before processing for paraffin embedding. Briefly, the membranes were removed from the inserts and prepared in Tissue Clear (Sakura, Osaka, Japan) for paraffin wax embedding using the Excelsior AS Tissue processor (ThermoFisher Scientific). Paraffin embedded sections (4 μm) were cut onto SuperFrost Plus slides (ThermoFisher scientific), dried over night at 37 °C, and then baked for 60 min at 60 °C. The sections were dewaxed in Tissue Clear and rehydrated through graded alcohols to water in an automatic slide stainer (Tissue-Tek Prisma, Sakura). Next, the sections were pretreated in Target Retrieval Solution, High pH (Dako, Glostrup, Denmark, K8004) in PT Link (Dako) for 20 min at 97 °C to facilitate antigen retrieval. The staining was performed according to the manufacturer’s procedure with EnVision G|2 Doublestain System Rabbit/Mouse (DAB+/Permanent Red) kit (Dako/Agilent K5361) on the Dako Autostainer. Following their soaking in wash buffer, we quenched endogenous peroxidase and alkaline phosphatase activity with Dual Endogenous Enzyme Block (Dako). Sections were then rinsed in wash buffer and incubated with primary antibody against Ki67 (MIB1 (Dako M7240)) diluted 1:300 for 40 min. The slides were rinsed before incubating in horseradish peroxidase (HRP) - polymer and 3,3′-Diaminobenzidine (DAB) to develop the stain. After a double stain block, the sections were incubated in antibody against cytokeratin 10 (Invitrogen #MA5-13705 diluted 1:100) for 60 min. After incubation in the mouse/rabbit linker, the sections were incubated in AP- polymer and the corresponding red substrate buffer with washing between each step. Tris-buffered saline (TBS; Dako K8007) was used throughout for the washing steps. The slides were lightly counterstained with hematoxylin, completely dried, and coverslipped. Appropriate negative controls were performed; both mouse monoclonal isotype control (Biolegend, San Diego, CA, USA) and omitting the primary antibody (negative method control).

### 2.10. Statistical Analysis

Statistical analysis was carried out in GraphPad Prism Software, version 7, using one-way ANOVA with Dunnet’s post-analysis. For normalized data, we used the Kruskal–Wallis test with Dunn’s post-analysis.

## 3. Results

### 3.1. Inhibition of cPLA2α Using AVX001 Resulted in a Balanced Reduction of Eicosanoids

**Figure A1:**
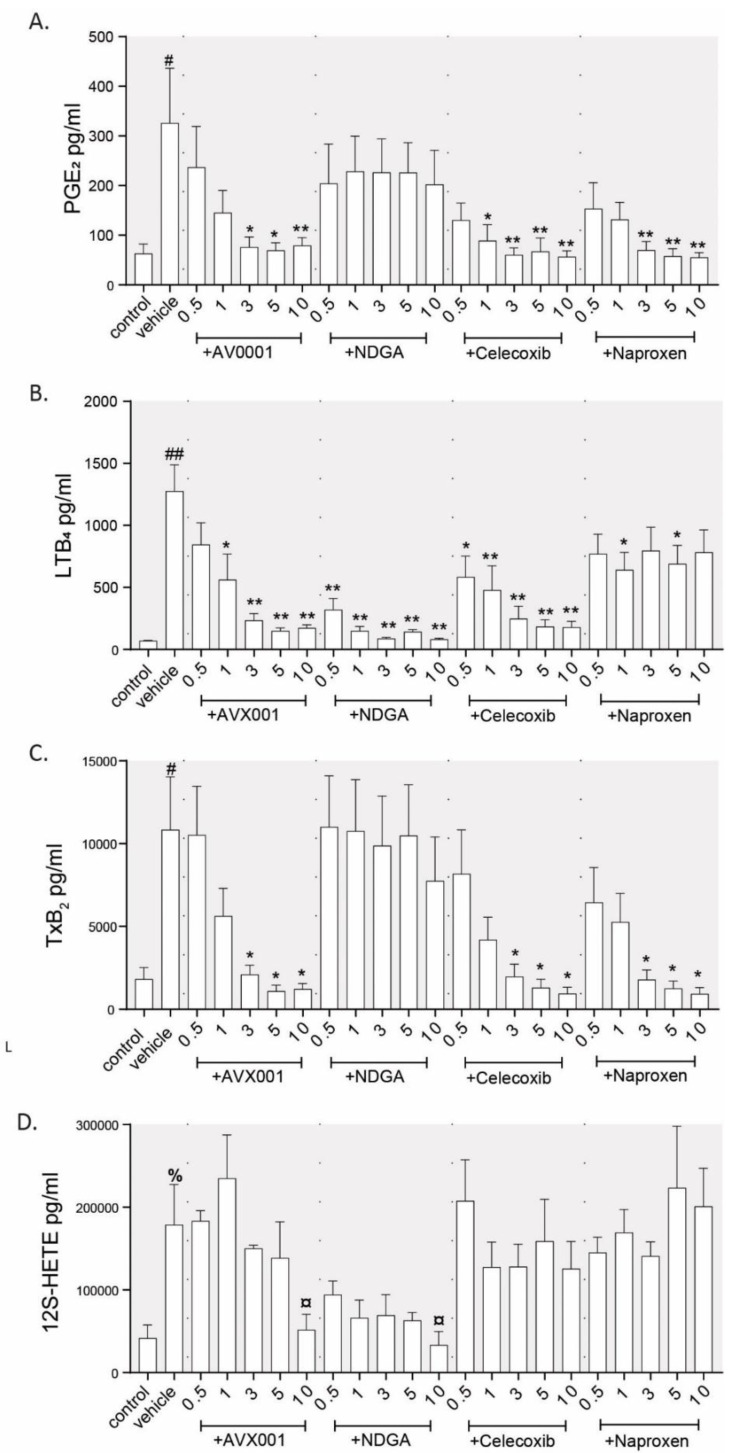
Inhibition of eicosanoid release in A23178-stimulated PBMC. Eicosanoid levels were measured in the supernatants of PBMC pre-incubated with vehicle, AVX001, naproxen, celecoxib, or NDGA in the range of 0.5–10 µM, and stimulated with A23178 (30 µM, 15 min). Data are the mean ± SEM for (**A**) PGE_2_, (**B**) LTB_4_, (**C**) TxB2, and (**D**) 12S-HETE from four individuals (PGE_2_, LTB_4_, TxB2) or three individuals (12S-HETE). Statistical significance was calculated using ANOVA with Dunnett’s post-analysis, whereby % *p* < 0.1, # *p* < 0.05, ## *p* < 0.01 versus unstimulated control and ¤ *p* < 0.1, * *p* < 0.05, ** *p* < 0.01 versus vehicle-treated control.

Several eicosanoids including PGE_2_, prostaglandin F_2_ (PGF_2_), LTB_4_, and 12S-HETE are known to be elevated in psoriatic lesions [20,21,22,23,49], and their direct roles in disease progression are supported by animal models of inflammatory skin disease [24,31,50,51]. Hence, it has been proposed that generating a balanced reduction in overall eicosanoid production is a promising therapeutic strategy [12,31]. To test whether the cPLA2α inhibitor AVX001 can normalize a broader range of eicosanoids than known COX- and LOX inhibitors, we treated PBMC with AVX001, the non-specific lipo-oxygenase (LOX) inhibitor nordihydroguaiaretic acid (NDGA), the cyclooxygenase (COX)-2 selective inhibitor celecoxib, or the dual COX1/COX2 inhibitor naproxen, and stimulated the mixture with the Ca^++^ ionophore A23178. The levels of PGE_2_, TxB_2_, LTB_4_, and 12S-HETE were assayed by ELISA and inhibition was calculated as a percentage of the A23178 stimulation. The maximum inhibition and concentration at which this was reached is presented in Table 1, and the absolute eicosanoid levels are available in Appendix A (Figure A1). We observed that AVX001 treatment dose-dependently inhibited the release of PGE_2_, TxB_2_, and LTB_4_. AVX001 also reduced 12S-HETE release at the maximum dose tested (10 µM), although it did not reach significance (*p* = 0.12, *n* = 3). As expected, NDGA dose-dependently inhibited the release of LOX metabolites LTB_4_ and 12S-HETE, but not COX metabolites PGE_2_ and TxB_2_. Celecoxib treatment gave a dose-dependent reduction of PGE_2_, TxB_2_, and LTB_4_ but did not inhibit 12S-HETE. The finding that the COX-2 selective inhibitor celecoxib reduced LTB_4_ release was surprising, however, it was consistent with the following study that demonstrated inhibition of 5-LOX but not 12- or 15-LOX by celecoxib in human blood [52]. Naproxen treatment, on the other hand, gave dose-dependent inhibition of the COX metabolites PGE_2_ and TxB_2_ without affecting LOX metabolites LTB_4_ or 12S-HETE. AVX001 treatment was therefore shown to result in a broader inhibition of eicosanoid release than the COX or LOX inhibitors tested, having a similar efficacy to reduce both COX and 5-LOX metabolites, and possibly also the 12-LOX metabolite 12S-HETE, albeit at a higher dose.

### 3.2. AVX001 Inhibited PGE_2_ Release in Response to Inflammatory Stimuli

AVX001 inhibited eicosanoid release from A23187-stimulated PBMC, however, the use of the Ca^++^ ionophore does not represent a physiological stimulus, and therefore it was important to confirm the effects using biologically relevant stimuli. For these experiments, we measured the PGE_2_ release. This was selected because PGE_2_ is one of the main eicosanoids produced in the skin, being released by epidermal keratinocytes, dermal fibroblasts, and immune cells. It has proinflammatory and immuno-modulatory properties and promotes keratinocyte proliferation (reviewed in [53]). We measured PGE_2_ levels in response to inflammatory stimuli and epidermal growth factor (EGF) and investigated the role of cPLA_2_α in these responses.

**Figure 1:**
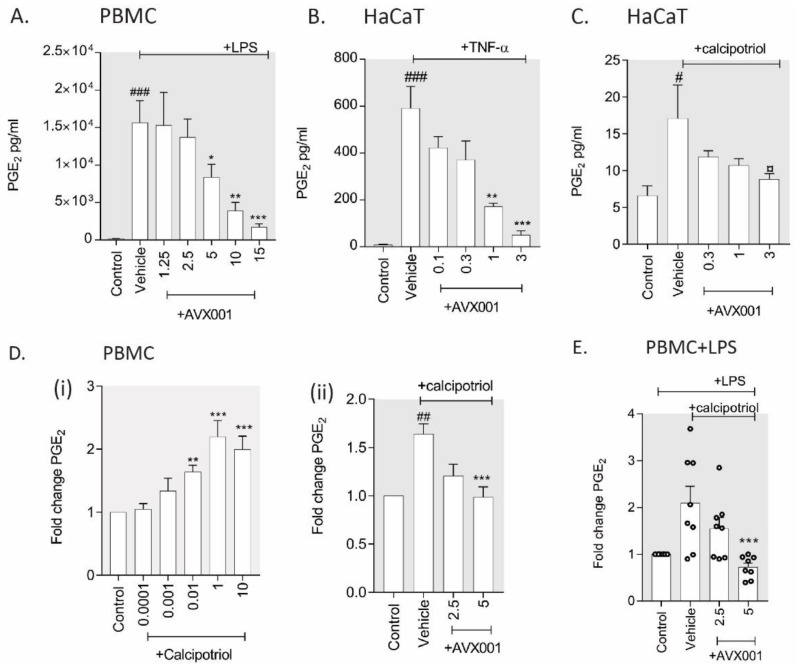
Effect of AVX001 on pro-inflammatory eicosanoid production. (**A**) PBMC were pre-incubated with AVX001 (µM) and stimulated with lipopolysaccharide (LPS) (10 ng/mL, 72 h). (**B**) Immortalized keratinocytes (HaCaT) were pre-incubated with AVX001 (µM) and stimulated with tumor necrosis factor (TNF)-α (10 ng/mL, 72 h). (**C**) HaCaT were pre-incubated with AVX001 (µM) and stimulated with calcipotriol (10 nM, 72 h). (**D**) (**i**) PBMC were treated with calcipotriol (µM) for 72 h, (**ii**) PBMC were pre-treated with AVX001 (µM) and stimulated with calcipotriol (10 nM, 72 h). (**E**) PBMC were pre-incubated with AVX001 (µM) and stimulated with calcipotriol (10 nM, 72 h) or vehicle in the presence of LPS (10 ng/mL). PGE_2_ levels in supernatants were determined by ELISA and reported as either pg/mL (**A**–**C**), or as the fold change in PGE_2_ levels over unstimulated (**D**) or LPS-stimulated (**E**) controls. Data are the mean ± SEM of ≥ 4 individuals (PBMC) or 3 replicates (HaCaT). Statistical significance was calculated by one-way ANOVA with Dunnett’s post-analysis, or, for normalized data, using the Kruskal–Wallis test with Dunn’s post-analysis; # *p* < 0.05, ## *p* < 0.01, or ### *p* < 0.005 versus unstimulated control and * *p* < 0.05, ** *p* < 0.01 versus vehicle-treated control.

To investigate the role of cPLA_2_α in the response to pro-inflammatory stimuli, we preincubated human PBMC or HaCaT with AVX001 and stimulated them with lipopolysaccharide (LPS) or tumor necrosis factor (TNF)-α, respectively. LPS stimulated PGE_2_ release in PBMC by an average of 91-fold. Pretreatment with AVX001 dose-dependently inhibited LPS-stimulated PGE_2_ release with an IC_50_ of 5 µM (Figure 1A). HaCaT released a low but detectable level of PGE_2_ that was stimulated by TNF-α treatment by an average of 67-fold. AVX001 also dose-dependently inhibited TNF-α-stimulated PGE_2_ release (Figure 1B). These data support a role for cPLA_2_α in mediating pro-inflammatory eicosanoid release from immune cells and keratinocytes, suggesting that AVX001 would have anti-inflammatory properties in the skin.

Skin toxicity is a common side-effect of a variety of drug types including the vitamin D receptor (VDR) agonist calcitriol and its analogue calcipotriol, both of which are used to treat psoriasis [54]. The pro-inflammatory effects of VDR agonists are suggested to involve PGE_2_ [55,56]. To investigate the role of cPLA_2_α in VDR agonist-mediated PGE_2_ release, we treated PBMC and HaCaT with calcipotriol in the absence or presence of AVX001. We observed a small but significant increase in PGE_2_ in HaCaT exposed to calcipotriol; pre-incubation with AVX001 reduced the stimulatory effect, reaching marginal significance (*p* < 0.1) at the highest dose used (3 μM) (Figure 1C). Exposure to calcipotriol also stimulated the release of PGE_2_ from PBMC by approximately twofold; pre-incubation with AVX001 (5 µM) blocked the calcipotriol-induced increase in PGE_2_ (Figure 1D). Calcipotriol also augmented the LPS-stimulated release of PGE_2_ in PBMC from a subset of individuals, although the high variability between the responses meant that this was not significant overall. In cases where PGE_2_ levels were increased, AVX001 (5 µM) inhibited the response (Figure 1E). Our data support the hypothesis that adverse skin reactions resulting from calcipotriol treatment could result from increased PGE_2_ and suggest that AVX001 may be useful to abrogate these reactions by limiting PGE_2_.

### 3.3. AVX001 Inhibited EGF-Stimulated Release of AA and PGE_2_.

**Figure 2:**
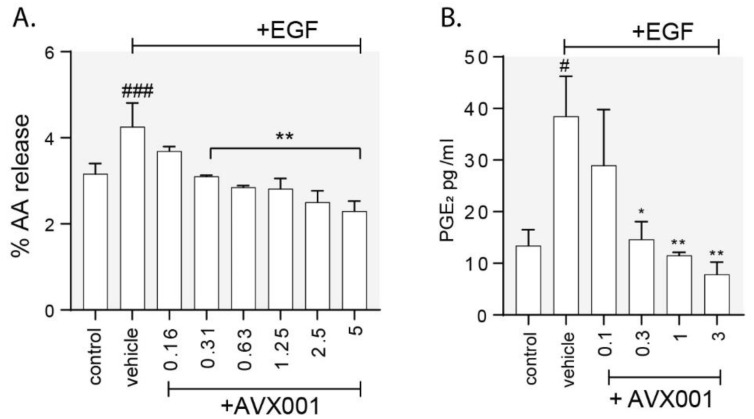
Effect of cPLA_2_α inhibition on epidermal growth factor (EGF)-dependent arachidonic acid (AA) and PGE_2_ release. (**A**) AA release was measured by the [3H]-arachidonic acid release assay in HaCaT treated with AVX001 and stimulated with EGF (100 ng/mL, 60 min). Data are the mean ± SD for three replicates from a representative experiment repeated twice. (**B**) PGE_2_ levels measured in the supernatant of cells preincubated with AVX001 at the indicated concentrations and stimulated with EGF (30 ng/mL, 24 h). Data are mean ± SEM for three replicates. Statistical significance was calculated by one-way ANOVA with Dunnett’s post-analysis; # *p* < 0.05, ### *p* < 0.005 versus unstimulated control and * *p* < 0.05, ** *p* < 0.01 versus vehicle-treated control.

Epidermal growth factor receptor (EGFR) activation is a well-known mitogenic signal for epidermal keratinocytes [57] and can activate cPLA_2_α via mitogen-activated protein kinase (MAPK)-dependent phosphorylation [3,58]. To investigate the effect of AVX001 on growth factor-mediated cPLA_2_α activation and eicosanoid release in keratinocytes, we stimulated HaCaT with EGF in the absence or presence of AVX001 and measured AA release and PGE_2_ levels. Consistent with activation of cPLA_2_α, EGF stimulated the release of both AA and PGE_2_ in HaCaT (Figure 2.) Treatment with AVX001 (≥ 0.3 µM) significantly and dose-dependently inhibited EGF-stimulated AA (Figure 2A) and PGE_2_ (Figure 2B) release.

### 3.4. AVX001 Inhibited Keratinocyte Proliferation

**Figure 3:**
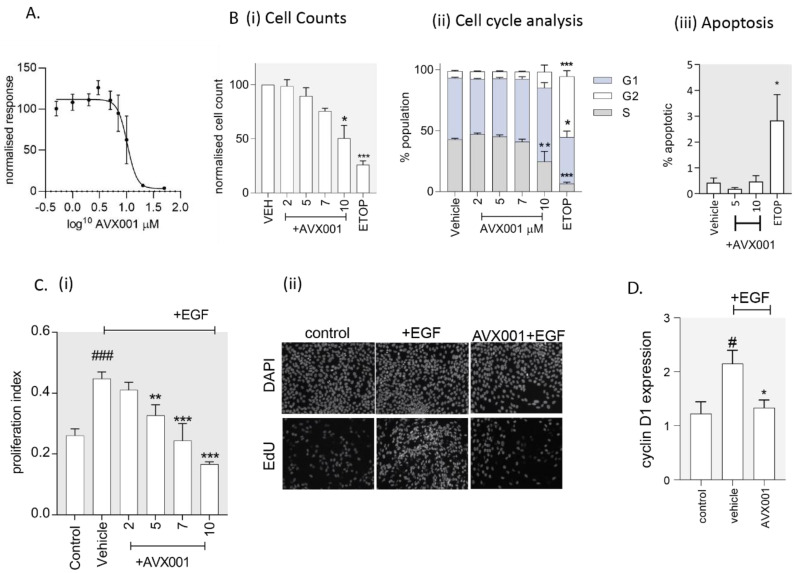
AVX001 inhibited proliferation in HaCaT monolayers. (**A**) Cell viability was measured using the resazurin assay in proliferating HaCaT treated with AVX001 for 24 h at the concentrations indicated. Measurements were normalized to the vehicle-treated control, and the mean ± SEM for three replicates is shown. (**B**) Automated microscopy and analysis of fluorescently labeled DNA, incorporated EdU, and caspase-3/7 activity was used to quantify (**i**) cell number, (**ii**) cell cycle distribution, and (**iii**) apoptosis in proliferating HaCaT treated with AVX001 for 24 h at the concentrations indicated. Data are mean ± SEM for ≥4 replicates. (**C**) (**i**) The proportion of cells in S-phase of the cell cycle (proliferation index) was determined by counting the total and EdU-positive nuclei in proliferating HaCaT pre-incubated with vehicle or AVX001 and stimulated with EGF (30 ng/mL, 24 h). Data are the mean ± SEM for three replicates from a representative experiment, repeated twice. (**ii**) Representative images showing 4′,6-diamidino-2-phenylindole (DAPI)-stained nuclei (DAPI) and fluorescently labelled EdU (EdU) for unstimulated HaCaT (control), and HaCaT stimulated with EGF (30 ng/mL, 24 h) in the absence (+EGF) or presence of 7 µM AVX001 (AVX001+EGF). (**D**) The relative expression of cyclin D1 measured by quantitative PCR in proliferating HaCaT pre-treated with AVX001 and stimulated with EGF (30 ng/mL, 4h). Data were normalized to the unstimulated control (control) and are the mean ± SEM from six replicates. Statistical significance was calculated by one-way ANOVA with Dunnett’s post-analysis, or, for normalized data, the Kruskal–Wallis test with Dunn’s post-analysis; # *p* < 0.05, ## *p* < 0.01, or ### *p* < 0.005 versus unstimulated control and * *p* < 0.05, ** *p* < 0.01, *** *p* < 0.005 versus vehicle-treated control.

We showed that inhibition of cPLA_2_α activity using AVX001 attenuated both AA and PGE_2_ release stimulated by the addition of exogenous growth factor in HaCaT. In fibroblasts, this signaling cascade was required for cell cycle progression [38]. We thus hypothesized that the treatment of keratinocytes with AVX001 could inhibit proliferation. To test this, we first treated actively proliferating HaCaT cells with AVX001 for 24 h and measured cell viability using resazurin as an indicator of metabolically active cells. We then quantified cell number, cell cycle distribution, and apoptosis using a high content image-based assay based on the fluorescent labeling and single-cell quantification of DNA, incorporated EdU, and caspase-3/7 activity, as detailed in Section 2.7. AVX001 inhibited HaCaT viability with an IC_50_ of 8.5 µM (Figure 3A). The reduced viability observed using 10 µM AVX001 was associated with a reduction in the total cell count (Figure 3B) (i) and a reduction in the proportion of the cells in S-phase (Figure 3B) (ii); there was no significant difference in the number of apoptotic cells (Figure 3B) (iii). The data also suggested an accumulation of cells in G1, although this finding was not statistically significant (*p* = 0.06). As a positive control, cells were treated with etoposide, a chemotherapy drug known to block cell cycle progression in HaCaT cells [59,60,61]. As expected, treatment with the etoposide (10 µM) caused a decrease in cell numbers that, in contrast to AVX001, was associated with an accumulation of cells in G2/M, and an increased proportion of apoptotic cells, consistent with its known effect as a blocker of the G2/M transition [62,63].

Treatment with EGF for 24 h did not significantly impact cell viability (not shown), but rather led to an increase in the proportion of cells in S-phase of the cell cycle (proliferation index), which was inhibited by AVX001 (≥5 µM) (Figure 3C). Progression of the cell cycle from G1 to S-phase in response to growth factors is typically associated with increased levels of cyclin D1 [64]. Following 4 h of EGF treatment, cyclin D1 transcript levels were increased approximately twofold in HaCaT, and this was inhibited by AVX001 (5 µM) (Figure 3D). These findings support cPLA_2_α as a target for inhibiting growth factor-dependent proliferation by halting cells in G1.

**Figure 4:**
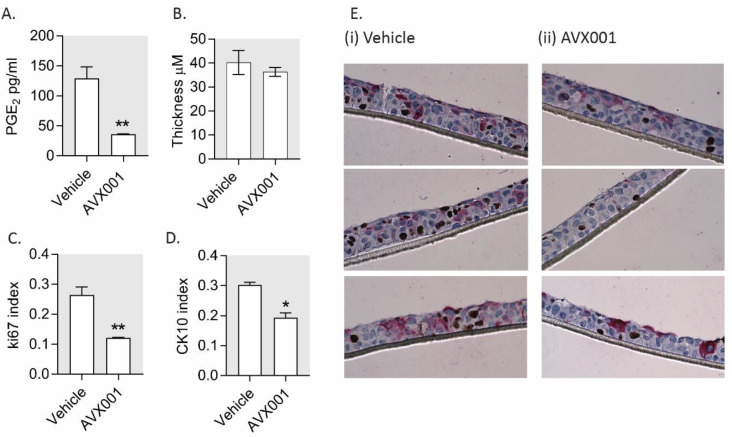
AVX001 inhibited proliferation in a stratified epithelium. HaCaT grown on porous collagen-coated membranes were maintained at the air–liquid interface for 12 days with exposure to exogenous EGF (1 ng/mL) in the absence or presence of AVX001 (5 µM). (**A**) PGE_2_ levels measured in the supernatants at day 12 by ELISA. (**B**–**D**) Immunohistochemistry using anti-Ki-67 MIB1 (DAB+) and anti-cytokeratin (CK)10 (Permanent Red) antibodies with hematoxylin counterstain. Brightfield images were taken at 40X magnification and used to quantify (**B**) the thickness (stratification) of the cultures, (**C**) the proportion of proliferating cells (proliferation index = Ki-67-positive cells/total cells), and (**D**) the proportion of CK10-positive cells (CK10 index = CK10 positive cells/total cells.) Typically, >20 images were collected at 40X magnification per replicate, with data being the mean ± SEM for three replicates. Statistical significance was calculated by one-way ANOVA with Dunnett’s post-analysis; * *p* < 0.05, ** *p* < 0.01, *** *p* < 0.005 versus vehicle-treated control. (**E**) Three representative images are shown for (i) vehicle and (ii) AVX001-treated cultures.

AVX001 inhibited cell proliferation in HaCaT grown in monolayers; however, experiments performed in 3D culture systems are often considered to have more physiological relevance. HaCaT retain the ability to stratify and differentiate in culture [65,66], and this is dependent on the presence of exogenous growth factors [67]. To test whether AVX001 is an effective inhibitor of proliferation in stratified keratinocytes, we cultured HaCaT at the air–liquid interface for 12 days in the absence or presence of AVX001. We measured PGE_2_ levels and the thickness of the stratified epithelia. The proportions of proliferating and differentiating cells were determined based on Ki-67 and cytokeratin (CK) 10 positivity, respectively. AVX001-treated cultures had reduced levels of PGE_2_, indicating the compound maintains the ability to suppress PGE_2_ levels in stratified cultures (Figure 4A.) There was no significant difference in the thickness of the cultures (Figure 4B,E), however, immunohistochemical analysis of Ki67 showed strikingly fewer proliferating cells in the AVX001-treated cultures compared to the vehicle-treated controls (Figure 4C,E). This was not, however, accompanied by an increase in the proportion of CK10-positive cells, as might be expected, but rather by an accompanying reduction in the proportion of CK10-positive cells (Figure 4D,E). These findings give support for cPLA_2_α being primarily a regulator of keratinocyte proliferation.

## 4. Discussion

In this study, we investigated the effects of the cPLA_2_α inhibitor AVX001 on inflammatory eicosanoid release and epidermal proliferation to understand its mode of action for treating psoriatic skin disease.

We demonstrate for the first time that AVX001 can significantly and dose-dependently suppress the production of both COX and LOX AA metabolites in stimulated human PBMC. The findings are consistent with the use of the cPLA_2_α inhibitors pyrrophenone and WAY-196025, which similarly inhibited both PGE_2_ and LTB_4_ release from PBMC stimulated with A23178 [68,69]. Our data thus support the fact that targeting the cPLA_2_α enzyme results in a balanced suppression of inflammatory eicosanoid release.

We further demonstrate the inhibitory effect of AVX001 on eicosanoid released in response to pro-inflammatory stimuli. The Toll-like receptor (TLR) 4 agonist LPS induced PGE_2_ release from PBMC, which was inhibited by AVX001. Our data using human PBMC supports the previously described involvement of cPLA_2_α in LPS-stimulated PGE_2_ production in THP-1 monocytes [35,70] reported to result from the induction of both the levels and activity of cPLA_2_α [5]. TNF-α is a pro-inflammatory cytokine and a key contributor to the pathogenesis of psoriasis [71]. TNF-α induced a robust production of PGE_2_ in HaCaT, which was inhibited by AVX001. These findings support cPLA2α as a mediator of the pro-inflammatory effects of TNF-α, as proposed by Sjursen et al. [36], however, while we demonstrated TNF-α-induced PGE_2_ production, Sjursen et al. reported that 6 h treatment with TNF-α preferentially induced HETE and not PGE_2_ production in HaCaT. It is therefore likely that stimulation of PGE_2_ release involves additional transcriptional upregulation of COX pathway enzymes in addition to cPLA_2_α activation in these cells, as demonstrated by Seo et al. [72].

Calcipotriol is a topical therapeutic for psoriasis and is known to cause skin irritation [54]. We demonstrate that calcipotriol stimulates the release of PGE_2_ both in PBMC and keratinocytes, which is in agreement with the following studies [55,56,73] and further supports the involvement of VDR/PGE_2_ signaling in drug-induced skin toxicity, as proposed by Shah et al. [56]. The mechanism by which calcipotriol stimulates PGE_2_ production is unclear. In AVX001-treated cells, calcipotriol was unable to stimulate PGE_2_ release, implicating that cPLA2α activation is required. This is in contrast to studies in keratinocytes by Ravid et al. [55], who suggest that the upregulation of COX-2 as opposed to increased AA production is responsible for the stimulation of PGE_2_ production. Doroudi et al. [74] present a VDR-independent mechanism for activation of cPLA_2_α by calcitriol via Ca^2+^/calmodulin-dependent protein kinase II (CAMKII)-dependent phosphorylation. It will be interesting to determine how PGE_2_ is regulated by calcipotriol in PBMC and keratinocytes and whether direct activation of cPLA_2_α by CAMKII is involved. For the treatment of psoriasis, calcipotriol is commonly combined with the potent corticosteroid betamethasone dipropionate (Daivobet), resulting in improved efficacy and tolerance [75]. This poses the possibility that the use of AVX001 could be an interesting non-steroidal alternative combination partner for reducing inflammation and improving tolerance to calcipotriol.

Our finding that EGF-stimulated PGE_2_ release in keratinocytes is reduced by inhibition of cPLA_2_α is in line with several reports linking EGF stimulation with AA release [76,77,78,79]. Furthermore, Naini et al. describe a requirement for intact cPLA_2_α/PGE2 signaling in growth factor-dependent cell cycle progression in both mouse embryonic fibroblasts (MEFs) and mesangial cells [38]. Collectively, this puts regulation of the cPLA_2_α enzyme, by means of its level and activity, in a central position to modulate growth factor-dependent responses. Thus, cPLA_2_α may control both inflammatory and mitogenic processes, which are hallmarks of the pathogenesis of psoriasis.

We further show that treatment with AVX001 inhibits EGF-stimulated S-phase entry and reduces the proliferation of HaCaT keratinocytes grown both in monolayers and stratified cultures. Our findings are in agreement with the established role of cPLA_2_α and eicosanoid signaling molecules as drivers of proliferation in several cancerous and non-cancerous cell types (reviewed in [80] and [81]). The described role of PGE_2_ as an autacoid growth factor [82,83] and effector of EGF responses in keratinocytes [84] make it a good candidate for mediating the effects of cPLA_2_α inhibition on keratinocyte proliferation. Knockout of the PGE_2_ receptor, EP2, also supports a role for PGE_2_ in regulating keratinocyte proliferation [85,86]. However, PGE_2_ is certainly not the only candidate, and a weakness of this study was our focus on the effects of AVX001 on AA metabolites. It is likely that cPLA_2_α inhibition with AVX001 would also suppress the production of LPC and its metabolites, e.g. platelet-activating factor (PAF). Like the eicosanoids, PAF has pro-inflammatory and proliferative effects in the epidermis [46,87,88], and PAF inhibition was found to suppress psoriasis-like skin disease progression in mice [89]. In future studies, it will therefore be important to determine whether AVX001 can also suppress the formation of LPC metabolites, as well as to determine which lipid mediators are the most critical effectors of keratinocyte proliferation under conditions of chronic inflammation.

## 5. Conclusions

In summary, we show that inhibition of cPLA_2_α with AVX001 inhibits eicosanoid release from primary human PBMC, limits the release of PGE_2_ in response to inflammatory mediators and EGF, and inhibits the proliferation of keratinocytes by preventing S-phase entry in response to growth factor stimulation. These findings suggest that the therapeutic mode of action of AVX001 in psoriasis could depend both on reducing inflammatory eicosanoid production and on inhibition of the hyperproliferative state of keratinocytes. We additionally propose that AVX001 could improve tolerance to calcipotriol, which could be relevant for developing a combination therapy to treat psoriasis.

## Figures and Tables

**Table 1:** Inhibition of eicosanoid release in A23178-stimulated peripheral blood mononuclear cells (PBMC). Eicosanoid levels were measured in the supernatants of PBMC pre-incubated with AVX001, naproxen, celecoxib, or nordihydroguaiaretic acid (NDGA) in the range of 0.5-10 µM and stimulated with A23178 (30 µM, 15 min). The percentage inhibition of the A23178-stimulated release was calculated for each eicosanoid and is shown as the mean ± standard error of the mean (SEM) for *n* = 4 (prostaglandin E_2_ (PGE_2_)), *n* = 4 (leukotriene B_4_ (LTB_4_)), and *n* = 3 (12S-hydroxyeicosatetranoic acid (HETE)) individuals. The inhibitor concentration at which maximal inhibition was reached is given below. Statistical significance was calculated using Kruskal–Wallis tests with Dunn’s post-analysis; * *p* < 0.05, ** *p* < 0.01. A graphical presentation of absolute eicosanoid levels is available in Figure A1.

Inhibitor	PGE_2_	6-keto PGF_*1*_α	LTB_4_	TxB_2_	12S-HETE
**AVX001**	93 ± 9 5 µM *	114 ± 16 5 µM	95 ± 6 5 µM *	106 ± 3 5 µM *	90 ± 6 10 µM
**Naproxen**	96 ± 6 5 µM **	102 ± 18 5 µM	48 ± 7 5 µM	106 ± 2 5 µM **	−18 ± 24 10 µM
**Celecoxib**	97 ± 5 5 µM *	108 ± 20 5 µM	91 ± 3 5 µM *	107 ± 2 5 µM **	42 ± 35 10 µM
**NDGA**	49 ± 7 10 µM	34.3 ± 7 10 µM	99 ± 1 10 µM *	42 ± 12 10 µM	105 ± 1 10 µM *
